# Supplementary material for: End Sequence Analysis Toolkit (ESAT) expands the extractable information from single-cell RNA-seq data
Source: Genome Res. 2016 Oct;26(10):1397–410. doi: 10.1101/gr.207902.116 (PMC5052061; doi:10.1101/gr.207902.116)
Supplement: Supplemental Material [file supp_gr.207902.116_Supp_Materials.tar.gz › ESAT-esat_v0.1/doc/html/classbroad_1_1pda_1_1seq_1_1rap_1_1_count_probes.html]

ESAT: broad.pda.seq.rap.CountProbes Class Reference


ESAT


- Main Page
- Related Pages
- Packages
- Classes
- Files

- Class List
- Class Index
- Class Hierarchy
- Class Members

All Classes Namespaces Files Functions Variables Enumerator Pages

- broad
- pda
- seq
- rap
- CountProbes

Static Public Member Functions |
Public Attributes |
Static Public Attributes |
Protected Member Functions |
List of all members

broad.pda.seq.rap.CountProbes Class Reference

Inheritance diagram for broad.pda.seq.rap.CountProbes:

[legend]

Collaboration diagram for broad.pda.seq.rap.CountProbes:

[legend]

|  |  |
| --- | --- |
| Static Public Member Functions | |
| static void | main (final String[] args) |
|  | |

|  |  |
| --- | --- |
| Public Attributes | |
| File | INPUT |
|  | |
| File | OUTPUT |
|  | |
| File | STATS |
|  | |
| Integer | T7BASES = 26 |
|  | |
| Integer | OVERLAP = 5 |
|  | |

|  |  |
| --- | --- |
| Static Public Attributes | |
| static final int | ORIGINAL\_ARRAY\_LEFT = 30 |
|  | |
| static final int | ORIGINAL\_ARRAY\_RIGHT = 20 |
|  | |
| static final int | MAIN\_ARRAY\_LEFT = 30 |
|  | |
| static final int | MAIN\_ARRAY\_RIGHT = 30 |
|  | |
| static final int | SIMPLE\_ARRAY\_LEFT = 15 |
|  | |
| static final int | SIMPLE\_ARRAY\_RIGHT = 15 |
|  | |
| static final int | ARRAY\_140105 = 17 |
|  | |
| static final int | MRNA\_ARRAY\_LEFT = 40 |
|  | |
| static final int | MRNA\_ARRAY\_RIGHT = 40 |
|  | |

|  |  |
| --- | --- |
| Protected Member Functions | |
| int | doWork () |
|  | |

## Detailed Description

Program to find read pairs in which one of the pairs aligns to at least part of the qPCR tag sequence of the probe. Input SAM files have been aligned to the full probe sequences (including the target sequence, e.g. of Xist).

Author
:   engreitz

## Member Function Documentation

|  |  |  |  |  |
| --- | --- | --- | --- | --- |
| int broad.pda.seq.rap.CountProbes.doWork | ( |  | ) |  |

protected

|  |  |  |  |  |  |
| --- | --- | --- | --- | --- | --- |
| static void broad.pda.seq.rap.CountProbes.main | ( | final String[] | *args* | ) |  |

static

Stock main method.

Parameters
:   |  |  |
    | --- | --- |
    | args | main arguments |

## Member Data Documentation

final int broad.pda.seq.rap.CountProbes.ARRAY\_140105 = 17

static

File broad.pda.seq.rap.CountProbes.INPUT

final int broad.pda.seq.rap.CountProbes.MAIN\_ARRAY\_LEFT = 30

static

final int broad.pda.seq.rap.CountProbes.MAIN\_ARRAY\_RIGHT = 30

static

final int broad.pda.seq.rap.CountProbes.MRNA\_ARRAY\_LEFT = 40

static

final int broad.pda.seq.rap.CountProbes.MRNA\_ARRAY\_RIGHT = 40

static

final int broad.pda.seq.rap.CountProbes.ORIGINAL\_ARRAY\_LEFT = 30

static

final int broad.pda.seq.rap.CountProbes.ORIGINAL\_ARRAY\_RIGHT = 20

static

File broad.pda.seq.rap.CountProbes.OUTPUT

Integer broad.pda.seq.rap.CountProbes.OVERLAP = 5

final int broad.pda.seq.rap.CountProbes.SIMPLE\_ARRAY\_LEFT = 15

static

final int broad.pda.seq.rap.CountProbes.SIMPLE\_ARRAY\_RIGHT = 15

static

File broad.pda.seq.rap.CountProbes.STATS

Integer broad.pda.seq.rap.CountProbes.T7BASES = 26

---

The documentation for this class was generated from the following file:

- java/broad/pda/seq/rap/CountProbes.java


---

Generated on Fri Aug 15 2014 15:11:03 for ESAT by  

 1.8.7
